# Prognostic value of perioperative NT-proBNP after corrective surgery for pediatric congenital heart defects

**DOI:** 10.1186/s12887-019-1830-y

**Published:** 2019-12-16

**Authors:** Fangqin Lin, Lingling Zheng, Yanqin Cui, Weidan Chen, Ramit Kumar Gupta, Huixian Li, Xinxin Chen, Huimin Xia, Huiying Liang

**Affiliations:** 1 Institute of Pediatrics, Guangzhou Women and Children’s Medical Center, Guangzhou Medical University, Guangzhou, China; 2 Cardiac Intensive Care Unit, Heart Center, Guangzhou Women and Children’s Medical Center, Guangzhou Medical University, Guangzhou, China; 3 Department of Pediateric Surgery, Guangzhou Women and Children’s Medical Center, Guangzhou Medical University, Guangzhou, China

**Keywords:** NT-proBNP, Congenital heart defects, Repeated measures, Prognosis

## Abstract

**Background:**

It is critically important to assess the prognostic value of NT-proBNP in the form of repeated measures among children undergoing surgery for congenital heart defects (CHD). The aim of the present study is to assess the value of repeated perioperative NT-proBNP in evaluating the time dependent and temporal trajectory in prognostics diagnosis during the perioperative period in a large series of children with CHD.

**Methods:**

Repeated measures of NT-proBNP from 329 consecutive children with CHD were obtained before and 1, 12, and 36 h after surgery, respectively. For fully utilizing longitudinal characteristics, we employed parallel cross-sectional logistic regression, a two stage mixed effect model and trajectories over time analysis to mine the predictive value of perioperative NT-proBNP on the binary outcome of prolonged intensive care unit (ICU) stay.

**Results:**

The two stage mixed effects model confirmed that both the mean NT-proBNP level (aOR = 1.46, *P* = 0.001) and the time trends had prognostic value on the prediction of prolonged ICU stay. In the fully adjusted logistic regression analyses based on gaussian distributions, “rapidly rising NT-proBNP” put the subjects at 5.4-times higher risk of prolonged ICU stay compared with “slowly rising” group (aOR = 5.40, *P* = 0.003).

**Conclusions:**

Comprehensive assessment of the time dependent and temporal trajectory in perioperative NT-proBNP, indicated by repeated measurements, can provide more accurate identification of children with higher risk of prolonged ICU stay after CHD surgery.

## Background

Congenital heart disease (CHD) accounts for nearly 1/3 of all major congenital anomalies (9 per 1000 live births) [1]. Although the improvements in surgical intervention have significantly benefited children with CHD, the incidence of adverse postoperative outcomes remains about 27% [2]. Thus, the identification of prognostic biomarkers for adverse outcome could confer multiple benefits. The guidelines published by the European Society of Cardiology(2016) recommend the clinical biomarkers Brain natriuretic peptide (BNP) and N-terminal pro-B-type natriuretic peptide (NT-proBNP) for diagnosis and prognosis of heart failure [3]. At present, some evidence have pointed out the prognostic value of BNP after surgical intervention for CHD in pediatrics [4–7]. Compared to BNP, NT-proBNP transportation is more stable, and its half-life in serum is longer than that of BNP [8]. Therefore, NT-proBNP becomes more important and better marker of cardiac strain over time.

Some researches pointed out the prognostic value of NT-proBNP after surgical intervention for CHD. Walsh [9] et al. found the preoperative NT-proBNP level was a significant predictor of duration of intensive care unit (ICU) stay and peak postoperative level was a predictor of the intensity of overall medical management. They attained NT-proBNP preoperatively and at 2, 12, 24, 48 and 72 h after surgery and just used related analysis to explore the relation between NT-proBNP level and outcome. Goei [10] et al. demonstrated that the difference in NT-proBNP between pre and postoperative was the strongest independent predictor of cardiac outcome. They applied multivariate Cox regression analyses to evaluate the relation between the change in NT-proBNP levels and the study end point. And Michael [11] et al. also assessed a reduction in NT-proBNP had a lower subsequent rate of cardiovascular death or HF hospitalization. Qu [12] et al. have confirmed that time-varying NT-proBNP level, particularly 1-h postoperative levels, had prognostic value on the prediction of outcome after surgery.

Compared with a single measurement, perioperative serial NT-proBNP measurements were more accurate in predicting postoperative adverse events, since it can take into account the hemodynamic stress caused by anesthesia and surgery [10, 13]. However, there is no general consensus on methods for examining data with a longitudinal repeated variable and non-time-varying outcome. Chronological order is often ignored in routine characteristics. In order to overcome the shortcomings from routine characteristics and in-depth dig the values of repeated measurement data, the use of time dependent assessment based on the approach of two stage mixed effects model for clinical trial analyses has recently gained broad supports [14, 15]. This model is more flexible than the previous approaches, and does not require sphericity of the covariance data structure [16]. And this model characterizes within-subject patterns of longitudinal measurements, and the association between features of the longitudinal measurements process and the duration of outcome events. Therefore, our study would use this model to assess NT-proBNP prognostic value of the time dependent effect.

In addition, temporal trajectory of NT-proBNP levels in patients has been confirmed to be the powerful diagnostic and prognostic indicator available in treatment of kidney or heart disease [17–19]. Trajectories over time allow to assess how the measured data has changed through two or more assessment points. In this study, we would combined the temporal trajectory analysis to dig more prognostic value from the repeated perioperative NT-proBNP.

Therefore, the aim of the present study is to assess the value of repeated perioperative NT-proBNP in evaluating the time dependent and temporal trajectory in prognostics diagnosis.

## Methods

### Subjects and data collection

This retrospective study was conducted at Guangzhou Women and Children’s Medical Center. We derived the data from the clinical system databases, 364 eligible consecutive patients undergoing CHD surgery at this hospital between June and December 2014. Patients younger than 18 years with CHD who underwent cardiac surgery were eligible for the study. Children who had preoperative arrhythmia (potentially malignant ventricular arrhythmias and high-degree atrioventricular block), rheumatic heart disease, infective endocarditis, myocarditis, pericardial disease, renal dysfunction, or neoplasms, or who could not be separated from cardiopulmonary bypass (CPB) were excluded. Thus, results from 329 cases were presented in this study, which with complete clinical information were included.

Plasma NT-proBNP levels were determined in each patient at preoperative, 1 h, 12 h and 36 h postoperatively, respectively. The NT-proBNP levels were measured with a commercially available fluorescence immunoassay (competitive Enzyme Immuno Assay; ReLIA II, Shenzhen, China) and Multi-Detection Microlpate Reader (VICTOR X5; PerkinElmer, Waltham, Mass). Clinical and biochemical data were collected retrospectively form the medical records, including patient demographics (age, weight, gender), the Risk Adjustment in Congenital Heart Surgery, version 1(RACHS-1) score, Cardiopulmonary bypass (CPB) duration, aorta cross clamp (ACC) time and follow-up measurements after surgery: the duration of Intensive Care Unit (ICU). The primary outcome measure was the prolonged ICU stay, as the short-term adverse outcome to estimate prognostic [20].. And the patients were divided two groups: ICU stay <= 3 days and ICU stay > 3 days according to the mean duration of ICU stay, which was 3.0 days with a median (interquartile range [IQR]) of (2–5) days (As shown in Additional file 1: Figure S1).

### Data analysis

Continuous variables were showed as mean and standard deviation and compared by using the 1-way analysis of variance for 2-group comparisons. Categorical variables were expressed as percentages and compared by using the Fisher exact test or the *X*^*2*^ test. NT-proBNP were compared between the two groups and was log-transformed to obtain normality. A parallel cross-sectional logistic regression model was built to evaluate the relation between NT-proBNP and prolonged ICU stay. Multivariate regression analyses were adjusted for cardiac risk factors and factors recognized to influence NT-proBNP levels: age, gender, RACHS-1, Weight, CPB time and ACC time.

Statistical analysis was performed using *R* software, version 3.3.2. The package of *nlme* was used for two stage mixed effects analysis. *Mclust* package was the optimal model for parameterized Gaussian mixture clustering by hierarchical clustering. The results of the logistic regression model analyses are presented as odds ratios (ORs) with 95% CIs. *P* < 0.05 considered statistically significant.

In addition, we applied two methods, time dependent assessment and trajectories over time, to explore whether the change of NT-proBNP could predict the postoperative recovery of children patients undergoing CHD surgery. For time dependent assessment, we proposed a two stage mixed effects model as followed. In stage 1, the longitudinally time-varying NT-proBNP level was first modeled using a linear mixed effects (LME) model. In the second stage, best linear unbiased predictor (BLUP) estimated of the random coefficients from LME model were used as predictors in a logistic regression model.

For trajectories over time, the statistical analysis also included two steps. First, subject-specific NT-proBNP levels were clustered based on Gaussian distributions with a predetermined number of clusters *(K)*. Correspondingly, each subject was grouped into one of the *K* clustered trajectories. Second, to examine the association between trajectory groups over 4 peri-operative assessment points and the binary outcome of prolonged ICU stay, we regressed *Y*_*i*_ on this trajectory index along with the covariates as:

1 $$ logit\left[\Pr \left({Y}_i=1|{C}_i,{Z}_i\right)\right]={\beta}_0+{C}_i\beta +{\overline{Z}}_i^T{\beta}_Z $$

where *C*_*i*_ is the clustering trajectory index, which may be more than one-dimensional when *K* > 2.

Second, we added clusters of NT-proBNP into the logistic regression model adjusted other risk factors to assess the effect of trajectories over time of NT-proBNP with the CHD pediatric prognostics after surgery.

## Results

### Study group characteristics

The patients’ clinical characteristics were listed in Table 1. Patients were grouped according to length of stay in the ICU: group1, 3 days or less (*n* = 181; 55.02%) and group 2, more than 3 days (*n* = 148; 44.98%). With the exception of gender, all the other clinical baseline characteristics showed significant differences between the two groups (all *P <* 0.001) (Table 2).

**Table 1:** Clinical characteristics of children undergoing congenital heart disease surgery

Characteristics	ICU stay <=3 days
Type of congenital heart disease	
Ventricular septal defect	163(49.59)
Atrial septal defect	34(10.47)
Total anomalous pulmonary venous connection	12(3.58)
Tetralogy of Fallot	24(7.44)
Transposition of the great arteries	23(6.89)
Pulmonary atresia	18(5.51)
Complete atrioventricular septal defect	14(4.13)
Coarctation	15(4.68)
Double outlet right ventricle	5(1.65)
Interrupted aortic arch	2(0.55)
Pulmonary stenosis	5(1.38)
Other types of CHD	14(4.13)
NT-proBNP level, pg/ml	
Before surgery	2744.28±5158.85
1h after surgery	2564.19±4363.56
12h after surgery	6797.01±6260.35
36h after surgery	4727.21±4321.34
Gender	
Female	117(35.56)
Male	212(64.44)
RACHS-1 score	
I/II	251(76.29)
III/IV	78(23.71)
Age, d	416.93±753.44
Weight, kg	7.05±5.39
Cardiopulmonary bypass time, min	84.87±45.47
Aortic crossclamp time, min	43.66±26.26

Categorical data are showed as n (%) responding group respectively. Continuous variables are showed as mean ± standard deviation. *ICU* Intensive Care Unit, *RACHS-1* Risk Adjustment for Congenital Heart Surgery-1

**Table 2:** Clinical characteristics of children with ICU stay time equal or less than 3 days and children with ICU stay time greater than 3 days undergoing CHD correction

Characteristics	ICU stay <=3 days	ICU stay >3 days	*p*
Patient number	181(55.02)	148(44.98)	
NT-proBNP level, pg/ml			
Before surgery	1214.07±2935.61	4615.68±6500.49	<0.001
1h after surgery	1045.29±1743.52	4421.76±5686.58	<0.001
12h after surgery	4714.70±5379.54	9343.61±6320.02	<0.001
36h after surgery	3076.17±2474.08	6746.39±5158.98	<0.001
Gender			
Female	68(37.57)	49(33.11)	0.42
Male	113(62.43)	99(66.89)	
RACHS-1 score			
I/II	165(91.16)	86(58.11)	<0.001
III/IV	16(8.84)	62(41.89)	
Age, d	573.62±900.76	225.31±450.45	<0.001
Weight, kg	8.56±6.38	5.20±2.94	<0.001
Cardiopulmonary bypass time, min	66.40±25.82	107.46±53.41	<0.001
Aortic crossclamp time, min	34.57±16.46	54.78±31.25	<0.001

Categorical data are showed as n (%) responding group respectively. Continuous variables are showed as mean ± standard deviation. *ICU* Intensive Care Unit. *RACHS-1* Risk Adjustment for Congenital Heart Surgery-1

**Fig. 1:**
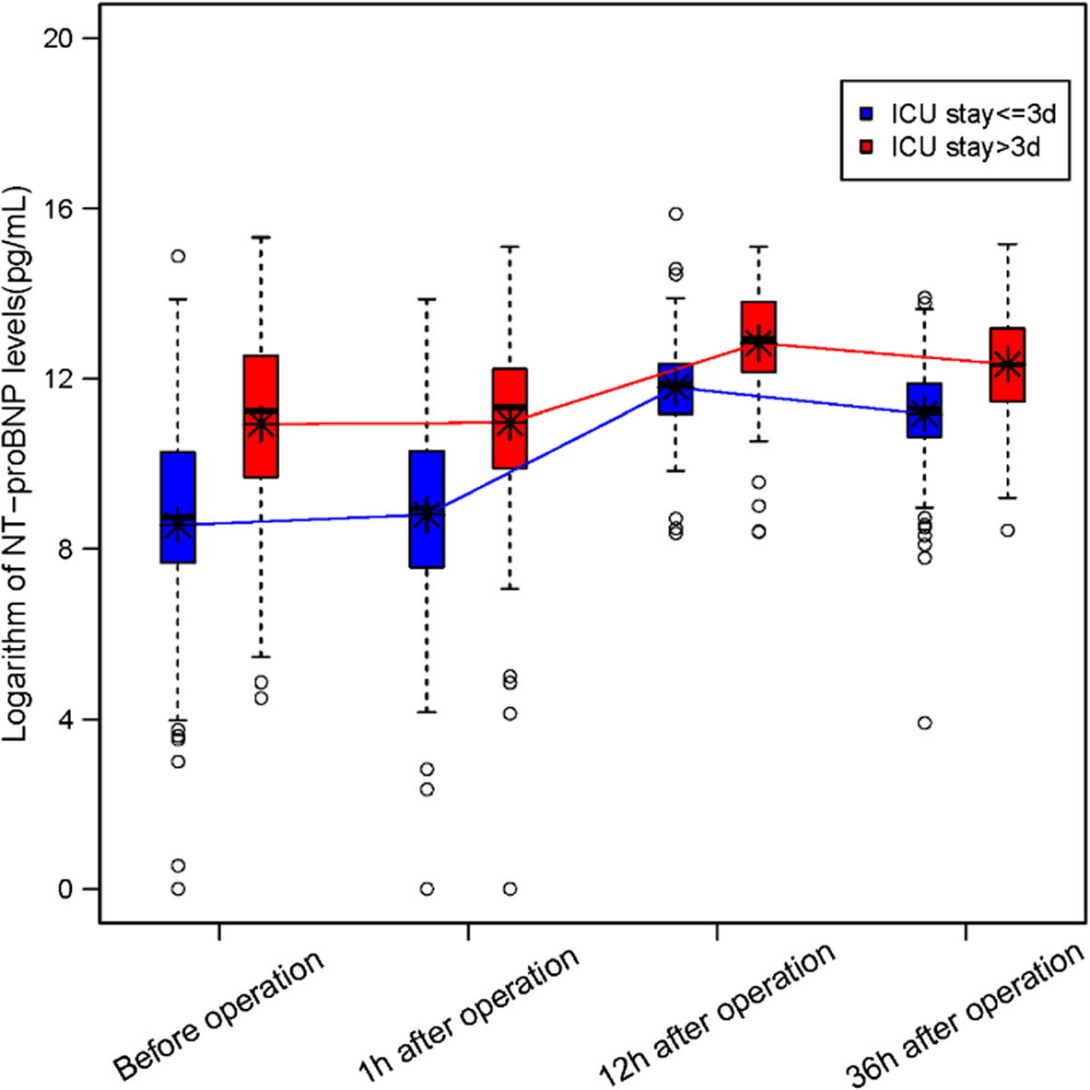
The comparisons of NT-proBNP levels between two groups at different time. Group 1(ICU stay <=3 days, blue color) and group 2(ICU stay > 3, red color) at before surgery, 1 hour, 12 hours, 36 hours after CHD surgery. Boxes show the interquartile range, and solid black lines show the median of NT-proBNP levels. Asterisks present the mean of NT-proBNP levels. All the NT-proBNP levels were logarithm transformed

The higher level of perioperative NT-proBNP was a potential prognostic factor for longer stay in ICU. In both groups, the level of postoperative NT-proBNP showed a rising trend regardless of preoperative condition and reached the peak at 12 h after operation (Fig. 1).

#### Prognostic value of time independent assessment of perioperative NT-proBNP on prolonged ICU stay

Parallel cross-sectional logistic regression models for prognostic value of time independent assessment are presented in Table 2. After adjustment for potential confounding factors, the values of NT-proBNP at each of the four times were non-statistically significant associated with prolonged ICU stay (Table 3).

**Table 3:** Prognostic value of time independent assessment of repeated perioperative NT-proBNP on prolonged ICU stay

Variables	Estimate	SE	OR^a^	95% CI	*p*
Method Parallel cross-sectional logistic regression models					
Before surgery	0.17	0.094	1.19	0.99 to 1.43	0.070
1h after surgery	0.083	0.10	1.09	0.89 to 1.34	0.42
12h after surgery	0.086	0.18	1.09	0.77 to 1.57	0.64
36h after surgery	0.24	0.17	1.27	0.91 to 1.81	0.16

*SE* standard error, *OR* odds ratio, *CI* confidence interval. ^a^-Models adjusted for age, gender, weight, cardiopulmonary bypass time, aorta cross clamp time, and RACHS-1 score

#### Prognostic value of time dependent assessment of perioperative NT-proBNP on prolonged ICU stay

We used two stage mixed effects model to further investigate the time dependent prognostic value of NT-proBNP. As exhibited in Table 4, patient-specific intercepts and visit-specific slopes were first extracted from Stage 1 and then continuously modeled in Stage 2. The results suggested that there was an association between the patient-specific intercepts and prolonged ICU stay(*β* = 0.38, SE = 0.11, aOR = 1.46, *P* = 0.001). Controlled factors mentioned in Table 4 and the time effect, this model showed the higher level of NT-proBNP predicted the higher risk of prolonged ICU stay. Furthermore, compared with before surgery, two effect estimates for both 12 h and 36 h postoperative time points were statistically significant (*P* = 0.01 and 0.004, respectively). This demonstrated that both the mean NT-proBNP exposure level and the time trends, as indicated by the patient-specific intercept and visit-specific slopes, respectively, were associated with prolonged ICU stay.

**Table 4:** Prognostic value of time dependent assessment of perioperative NT-proBNP on prolonged ICU stay (Two stage mixed effects model)

Variables	Estimate	SE	OR^a^	95% CI	*p*
The first submodel					
Intercept	10.74	0.53	4.60×10^4^	1.64×10^4^ to 1.29×10^5^	<0.0001
Time points					
Before surgery	Reference				
1h after surgery	0.15	0.12	1.16	0.93 to 1.46	0.19
12h after surgery	2.63	0.12	13.90	11.07 to 17.45	<0.0001
36h after surgery	2.07	0.12	7.89	6.29 to 9.91	<0.0001
Gender					
Female	Reference				
Male	-0.13	0.086	0.88	0.74 to 1.04	0.12
RACHS-1					
I/II	Reference				
III/IV	0.52	0.12	1.68	1.32 to 2.14	<0.0001
Age, d	-0.14	0.046	0.87	0.79 to 0.95	0.002
Weight, kg	-1.18	0.12	0.31	0.24 to 0.39	<0.0001
CPB time, min	0.41	0.10	1.51	1.23 to 1.85	<0.0001
ACC time, min	0.069	0.064	1.07	0.95 to 1.21	0.28
The second submodel					
NT-proBNP levels	0.38	0.11	1.46	1.19 to 1.84	0.001
Time points					
Before surgery	Reference				
1h after surgery	0.51	0.28	1.67	0.98 to 2.90	0.06
12h after surgery	-1.91	0.76	0.15	0.03 to 0.65	0.01
36h after surgery	1.59	0.55	4.92	1.72 to 14.89	0.004
Gender					
Female	Reference				
Male	0.09	0.19	1.09	0.76 to 1.60	0.64
RACHS-1					
I/II	Reference				
III/IV	0.87	0.36	2.38	1.20 to 5.02	0.02
Age, d	-1.08	3.18	0.34	6.7×10^-4^ to 1.82×10^2^	0.73
Weight, kg	1.03	0.92	2.81	0.45 to 17.25	0.26
CPB time, min	-2.33	1.75	0.097	0.003 to 3.56	0.18
ACC time, min	-0.69	3.17	0.50	0.001 to 258.16	0.83

*SE* standard error, *OR* odds ratio, *CI* confidence interval, *CPB* cardiopulmonary bypass, *ACC* aorta cross clamp, *RACHS-1* Risk Adjustment for Congenital Heart Surgery-1. ^a^-Models adjusted for age, gender, weight, cardiopulmonary bypass time, aorta cross clamp time, and RACHS-1 score

#### The prognostic value of perioperative NT-proBNP levels trajectories on prolonged ICU stay

**Fig. 2:**
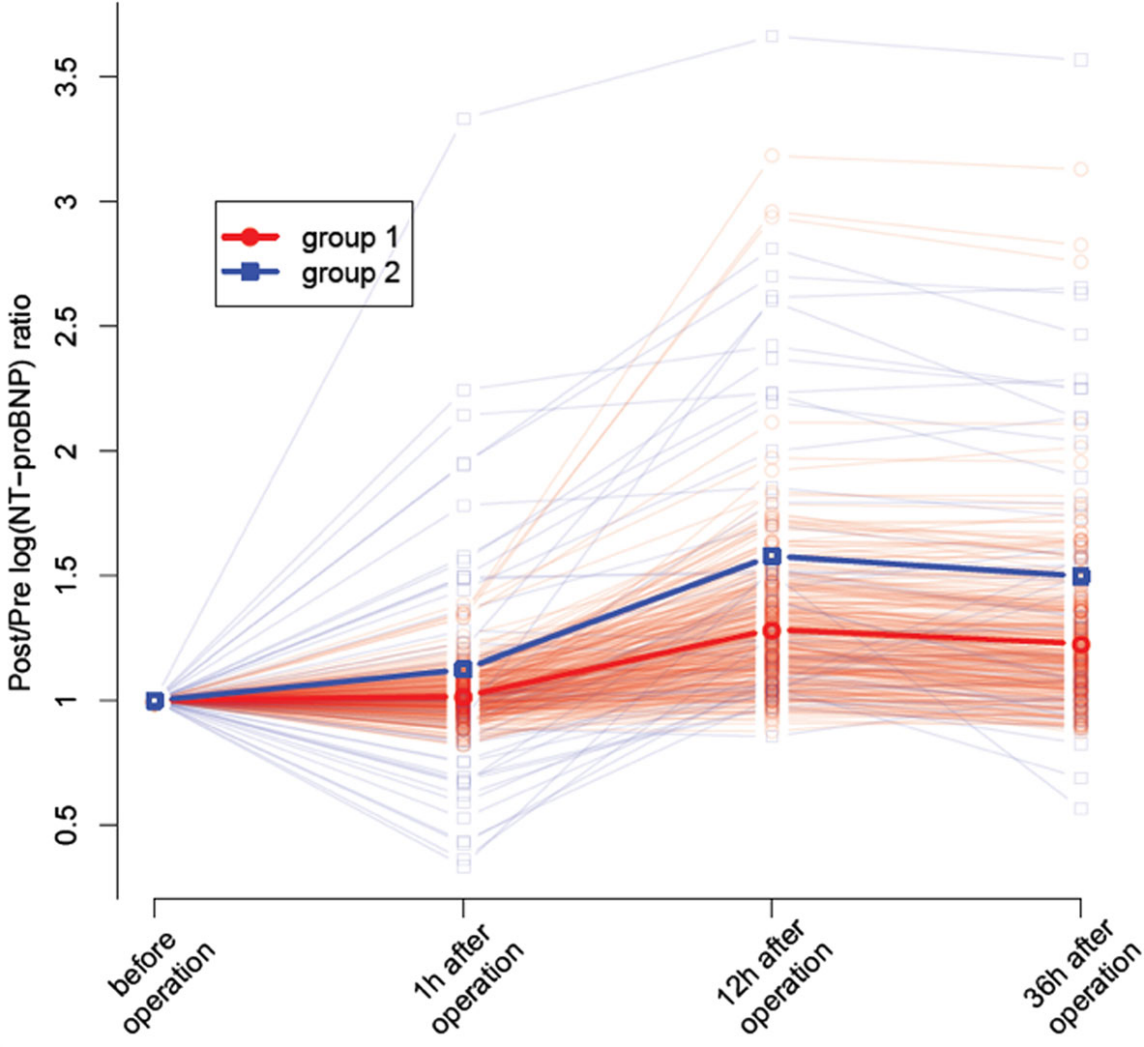
Comparisons of postoperative changes of NT-proBNP ratios over time between two clusters by Gaussian mixture clustering model. Red solid line represents mean of slowly rising group. Blue solid line represents mean of fast rising group. Semi-transparant lines represents ratio of NT-proBNP levels in every children with CHD undergoing cardiac surgery. The scale of y axis is log transformed

According to Bayesian information criterion (BIC), the number of clusters chose was two, based on Post−/Pre-(log NT-proBNP) ratio. The average ratios of the NT-proBNP levels within each cluster by time point were displayed in Fig. 2. The trajectory of NT-proBNP levels of cluster 1 elevated slowly over time, whereas rose rapidly in cluster 2. In detail, 87% of subjects fell into cluster 1 and 13% of them fell into cluster 2.

We then modeled each clustering index in relation to odds of suffering a prolonged ICU stay. As indicated in Table 5, compared with the odds for children in cluster 1, the odds of suffering a prolonged ICU stay for children in cluster 2 was 5.4 times (*β* = 1.67, SE = 0.56, aOR = 5.40, *P* = 0.003). It means that patients with rising rapidly NT-proBNP levels postoperative might have poorer postoperative recovery.

**Table 5:** Prognostic value of trajectories over time of repeated perioperative NT-proBNP on prolonged ICU stay

Variables	Estimate	SE	OR	95% CI	*p*
Intercept	-7.83	1.97	4.0×10^-4^	7.27×10^-6^ to 1.70×10^-2^	<0.0001
Trajectories over time					
Cluster 1	Reference				
Cluster 2	1.67	0.56	5.40	1.82 to 16.76	0.003
Gender					
Female	Reference				
Male	-0.12	0.31	0.89	0.48 to 1.62	0.70
RACHS-1					
I/II	Reference				
III/IV	0.46	0.45	1.59	0.66 to 3.84	0.30
Age, d	0.17	0.19	1.19	0.81 to 1.73	0.36
Weight, kg	-2.81	0.60	0.060	0.02 to 0.18	<0.0001
CPB time, min	2.51	0.42	12.33	5.68 to 29.54	<0.0001
ACC time, min	-0.46	0.22	0.63	0.39 to 0.95	0.03

*SE* standard error, *OR* odds ratio, *CI* confidence interval, *CPB* cardiopulmonary bypass, *ACC* aorta cross clamp, *RACHS-1* Risk Adjustment for Congenital Heart Surgery-1. ^a^-Models adjusted for age, gender, weight, cardiopulmonary bypass time, aorta cross clamp time, and RACHS-1 score

## Discussion

The principal findings of this study are as follows: (1) the higher perioperative NT-proBNP level prolonged ICU stay for pediatric patients with CDH after surgery; (2) both the mean NT-proBNP exposure level and the time trends were associated with prolonged ICU stay; (3) patients with rising rapidly perioperative NT-proBNP level suffer higher risk of prolonged ICU stay.

Firstly, we carried out assessment of prognostic value within other influence factors (CPB time, ACC time, gender, age, weight and RACHS-1 score). Some studies have pointed out that CPB duration is an independent predictor of adverse outcomes after cardiac surgery [21]. CPB also influenced BNP/NT-proBNP levels proportionally, which was confirmed in our first sub-model of two stage mixed effects model. The inconsistencies among previous findings that prolonged ACC time significantly correlated with major post-operative adverse events [22], were due to strong correlation between CPB time and ACC time [23]. Here, we found the correlation coefficient was 0.84 (*P* < 0.001) in our results (Additional file 1: Figure S2). Regardless of the variable of CPB time, length of ACC time restored predictive ability correctly in multiple models (data not shown). RACHS-1 was created to compare in-hospital mortality for children undergoing CHD surgery and demonstrated available for prediction of ICU stay length [24]. To a certain extent, our results supported that, RACHS-1 risk category was positively correlated with length of ICU stay in two stage mixed effect model. Other variables, such as age and gender almost showed not significant association with adverse outcomes.

Increasing evidence pointed out that the change in NT-proBNP have been shown to play key roles as prognostic biomarkers in patient undergoing cardiac surgery for CHD correction [10, 25]. Similar results were reported that the higher NT-proBNP could predict the longer duration of ICU stay [9] . Our results found that children stay in ICU longer than 3 days showed higher level NT-proBNP at each time point, which confirmed NT-proBNP has predictive value in pediatric CHD patient undergoing surgery. Otherwise, our result showed the peak level of NT-proBNP at 12 h after surgery, which Jiangbo had discuss before [12]. Then we further developed a parallel cross-sectional logistic regression model with covariate, like age or gender, to analysis the predictive value of each time point. However, the result of this model showed that NT-proBNP levels at each time point had no significant effect on the incidence of prolonged ICU stay. According to previous study [11, 12], we assumed that the time effect might play a role. So, we chose two stage mixed effects model to investigate time dependent prognostic value of perioperative NT-proBNP. Results from this model indicated that the mean level of NT-proBNP, especially at the 12 h and 36 h postoperative time points, significantly correlated to duration of ICU stay. Surprisingly, compared to the level of before surgery, NT-proBNP levels at 12 h after surgery, the peak level after surgery, was negatively associated with outcome event. After considering the time effect of the NT-proBNP with prolong.

ed. ICU stay, the peak NT-proBNP level become negative effect with the risk of longer prolonged ICU. It suggested that it is not accurate enough to judge the prognosis of CHD children solely based on the indicators of a single time point, like the peak time point.

So we further used temporal trajectory analysis to dig more prognostic value of temporal trajectories of perioperative NT-proBNP levels in patients. Although NT-proBNP has been confirmed to be the powerful diagnostic and prognostic indicator available in treatment of kidney or heart disease [17–19], to our knowledge, this is the first study to assess the prognostic value of temporal trajectory of longitudinal NT-proBNP levels by clustering model in children undergoing cardiac surgery for CHD. When patients undergo surgical procedures, the NT-proBNP levels at specific time points was closely related to individual basal secretion, so using conventional characteristic directly to predict the incidence of heart defects was highly susceptible. In our study, the time trajectory of NT-proBNP was proven to be a better prognostic indicator, since it can reflect an acute change in volume state or left ventricular function that influenced subsequent risk of adverse event. Clinicians might could judge the prognosis of patients by considering the speed of changes of NT-proBNP to adjust their medical decision.

## Conclusions

In conclusion, compared with single measurement, repeated measures of perioperative NT-proBNP levels were regarded as a more valuable predictor of ICU stay time length for CHD children undergoing open heart operation. Both the mean NT-proBNP exposure levels and the time trends were associated with prolonged ICU stay, and patients with rising rapidly perioperative NT-proBNP level suffer higher risk of prolonged ICU stay. Further studies are needed to confirm how the time trajectory of NT-proBNP can be used in clinical practice to predict postoperative risk and if repeated measurements of perioperative NT-proBNP should be performed during postoperative management to identify CHD children at high risk of adverse events.

## Supplementary information

**Additional file 1. Figure S1.** The vioplot of duration of ICU stay among 329 patients. Black box shows the interquartile range, solid white point shows the median of NT-proBNP levels, and black lines extend out from the box are up and down whiskers lines. The external shape with pink color is the kernel density estimation, which presents the population distribution among ICU stay time. **Figure S2.** The dash line showed CPB duration strongly correlated with AACC time. Cardiopulmonary bypass (CPB) duration strongly correlated with Aortic cross-clamping (ACC) time. Dash line is the regression between CPB duration and ACC time. Correlation coefficient is 0.84 and *p* value is less than 0.001.

## Data Availability

Not applicable.

## Abbreviations

ACC: Aorta cross clamp
BLUP: Best linear unbiased predictor
BNP: Brain natriuretic peptide
CHD: Congenital heart defects
CPB: Cardiopulmonary bypass (CPB) duration
ICU: Intensive care unit
LME: Linear mixed effects
NT-proBNP: N-terminal pro-B-type natriuretic peptide
RACHS-1: the Risk Adjustment in Congenital Heart Surgery, version 1
