# Supplementary material for: Prognostic value of perioperative NT-proBNP after corrective surgery for pediatric congenital heart defects
Source: BMC Pediatr. 2019 Dec 16;19:497. doi: 10.1186/s12887-019-1830-y (PMC6913023; doi:10.1186/s12887-019-1830-y)
Supplement: Supplementary file 1 — Additional file 1. Figure S1. The vioplot of duration of ICU stay among 329 patients. Black box shows the interquartile range, solid white point shows the median of NT-proBNP levels, and black lines extend out from the box are up and down whiskers lines. The external shape with pink color is the kernel density estimation, which presents the population distribution among ICU stay time. Figure S2. The dash line showed CPB duration strongly correlated with AACC time. Cardiopulmonary bypass (CPB) duration strongly correlated with Aortic cross-clamping (ACC) time. Dash line is the regression between CPB duration and ACC time. Correlation coefficient is 0.84 and p value is less than 0.001. [file 12887_2019_1830_MOESM1_ESM.docx]

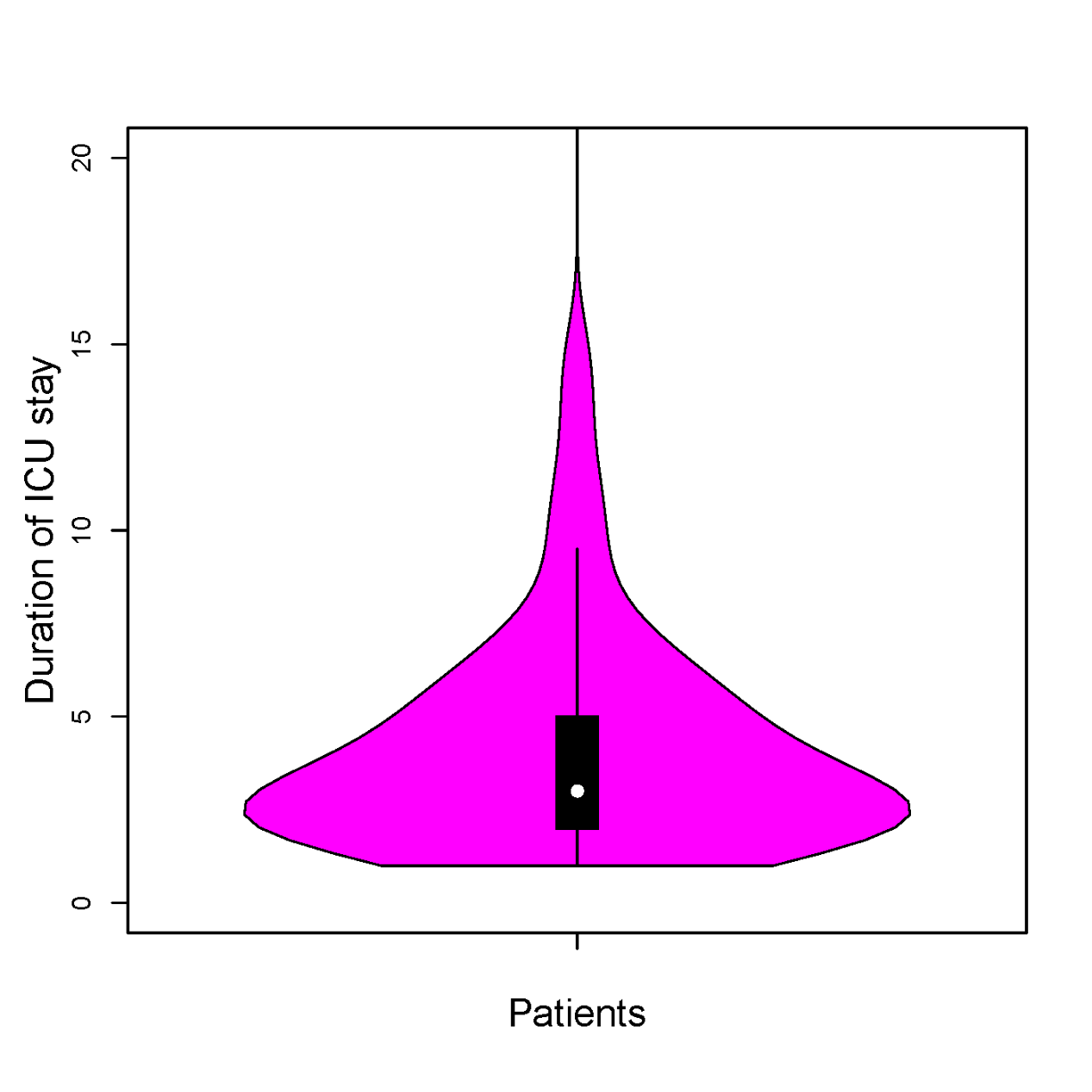


**Figure S1.** Vioplot of duration of ICU stay among 329 patients.

**Supplementary figure 1.** Vioplot of duration of ICU stay among 329 patients. Black box shows the interquartile range, solid white point shows the median of NT-proBNP levels, and black lines extend out from the box are up and down whiskers lines. The external shape with pink color is the kernel density estimation, which presents the population distribution among ICU stay time.


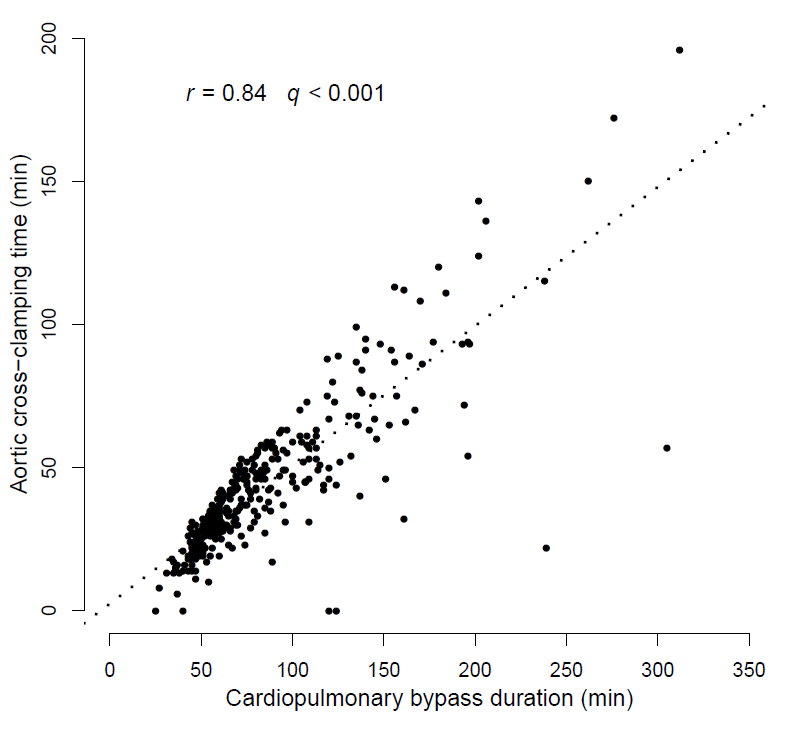


**Figure S2.** The dash line showed CPB duration strongly correlated with AACC time.

**Supplementary figure 2.** Cardiopulmonary bypass (CPB) duration strongly correlated with Aortic cross-clamping (ACC) time. Dash line is the regression between CPB duration and ACC time. Correlation coefficient is 0.84 and *p* value is less than 0.001.
